# Apocrine poroma: a case with multiple lesions^⋆^

**DOI:** 10.1016/j.abd.2023.08.019

**Published:** 2024-08-23

**Authors:** Natalia Scardua Mariano Alves, Bianca Cristina Dantas, Luana Rytholz Castro, Bethânia Cabral Cavalli Swiczar, Neusa Yuriko Sakai Valente

**Affiliations:** Department of Dermatology, Hospital do Servidor Público Estadual, São Paulo, SP, Brazil

*Dear Editor*,

Apocrine poroma is an extremely rare benign adnexal tumor. Traditionally, poromas are classified as eccrine. However, there are some reports describing poroid tumors with sebaceous, follicular, and apocrine differentiation.^1^, ^2^ The more recent identification and description of this group is possibly justified by the histological similarity between the ducts of these glands, if not identical, although the eccrine and apocrine glands are histologically different.^3^ In 1981, Grosshans et al.^3^ described, for the first time, poromas with sebaceous and apocrine differentiation, classifying this group as “infundibular adenomas”. Subsequently, new designations were suggested for the same histopathological findings, some of them being “adnexal poroma-like adenoma”;^4^ “sebocrine adenoma”, “apocrine-sebaceous analog”;^1^ and finally “apocrine poroma”.

Apocrine structures have the same embryonic origin as the pilosebaceous system and develop as a single unit. On the other hand, the eccrine gland and its duct develop from another epithelial bud.^4^, ^5^ Therefore, considering the common embryological origin of the follicular-sebaceous-apocrine unit, the term “apocrine poroma” has become established as the most appropriate term for adnexal tumors such as the one described here.

This is a case report of a little-known adnexal cutaneous tumor, which has clinical characteristics and histopathological signs of apocrine derivation. This is the second report of multiple apocrine poromas in a single patient.

A 64-year-old female patient had five asymptomatic lesions scattered across the trunk that had appeared slowly and progressively over the last two years. The patient did not use any medication, oral or topical, and did not undergo any previous procedure.

The physical examination showed erythematous-violaceous vegetative lesions, the largest measuring 1.5 cm in its largest diameter and the smallest measuring 0.5 cm, with irregular edges, a granular surface, and a slight projection on the mid-back region (2), on the upper lateral quadrant of the right breast (1), on the hypogastrium (1) and on the left flank (1; Fig. 1). Metastatic breast carcinoma, melanoma, and hemangioma were suggested as differential diagnoses.

**Figure 1:**
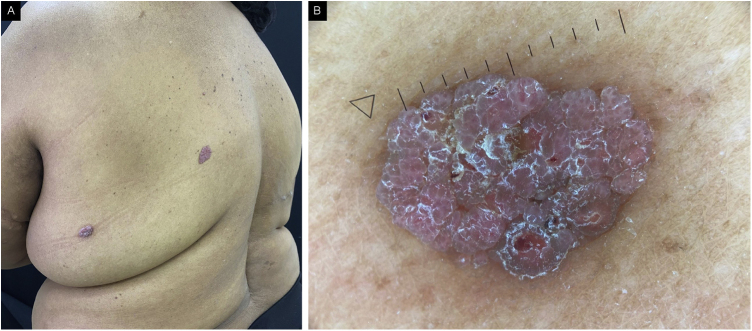
(A) Apocrine poromas on the back, clinical appearance. Erythematous-violaceous vegetative lesions with a granular surface and slight projection. (B) Apocrine poroma, dermoscopic presentation.

An incisional biopsy was then performed on two of the described lesions. Histopathology revealed the proliferation of basaloid cells without atypia, in anastomosing cords connected to the epidermis, with the formation of glandular lumens of varying sizes and papillary projections of the lining (Figure 2, Figure 3). The glandular lumens were lined by epithelium with decapitation secretion, characteristic of apocrine differentiation (Fig. 4).

**Figure 2:**
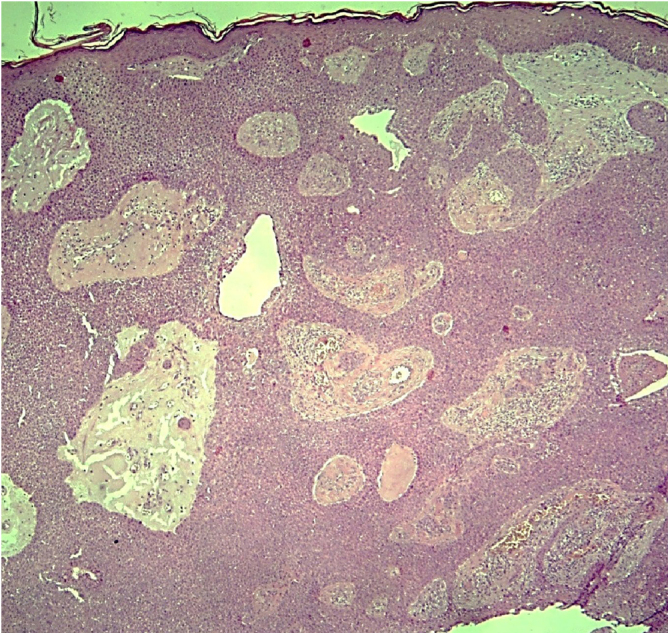
Apocrine poroma: proliferation of basaloid cells without atypia, in anastomosing cords connected to the epidermis with the formation of glandular lumens. (Hematoxylin & eosin, X40).

**Figure 3:**
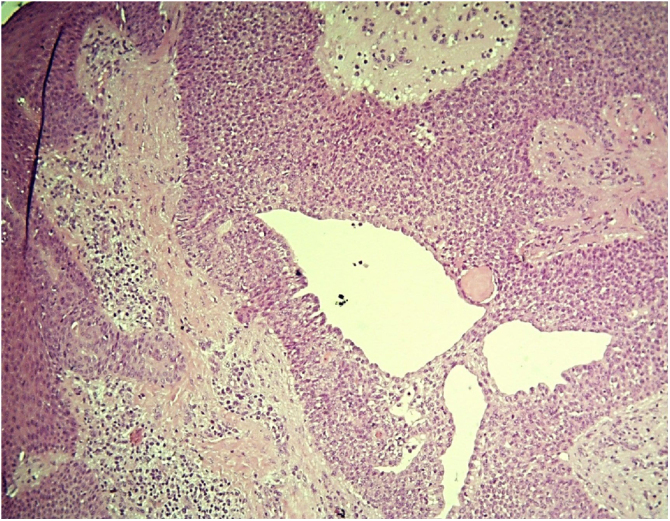
Apocrine poroma: proliferation of basaloid cells without atypia forming glandular lumens with papillary projections of the lining. (Hematoxylin & eosin, X100).

**Figure 4:**
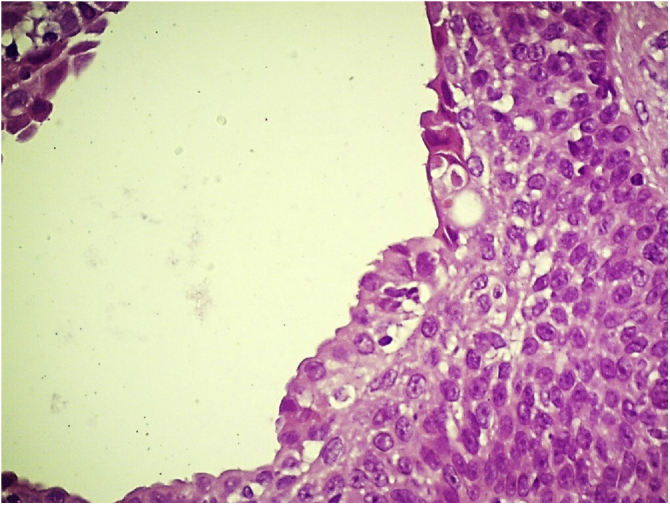
Apocrine poroma: Glandular lumen lined by apocrine epithelium with decapitation secretion. (Hematoxylin & eosin, X400).

Although isolated cases of apocrine poromas^5^, ^6^, ^7^ and some case reports of multiple eccrine poromas have been reported,^8^ to the best of the authors knowledge, this is the second report of multiple apocrine poromas in a single patient, the first having been described in 2015, in Japan.^9^

The microscopic analysis of the present case showed basaloid cells without atypia in anastomosing cords connected to the epidermis, with the formation of glandular lumens with decapitation secretion, which led to the diagnosis of poroma of apocrine origin. The presence of sebocytes and connection with the hair follicle was also identified in the tumor, which reinforces the apocrine differentiation. Immunohistochemistry was positive for epithelial membrane antigen (EMA) and negative for carcinoembryonic antigen (CEA).

Regarding malignant transformation, porocarcinoma with apocrine differentiation has not yet been described in the literature. However, Requena et al. reported that porocarcinomas may be continuous with the infundibula, indicating an apocrine origin. They further assume that, although the majority of poroid neoplasms are described as eccrine, the transdifferentiation of malignant poroid cells has progressed to such an extent that a distinction between eccrine and apocrine origin is not possible in the majority of cases. It is reasonable to assume that apocrine poromas can potentially undergo malignant transformation. For these reasons, all poromas, including those of apocrine derivation, must be completely excised.

Finally, it is worth reinforcing the importance of reporting cases of apocrine poromas, since as poromas and porocarcinomas have become increasingly recognized as also being of apocrine origin, it is possible that the clinical, histopathological and prognostic differences between tumors of eccrine and apocrine origin may become better known.

## Financial support

None declared.

## Authors’ contributions

Natalia Scardua Mariano Alves: Design and planning of the study; drafting and editing of the manuscript; collection, analysis and interpretation of data; critical review of the literature.

Bianca Cristina Dantas: Design and planning of the study; drafting and editing of the manuscript; collection, analysis and interpretation of data; critical review of the literature.

Luana Rytholz Castro: Design and planning of the study; drafting and editing of the manuscript; collection, analysis and interpretation of data; critical review of the literature.

Bethânia Cabral Cavalli Swiczar: Approval of the final version of the manuscript; effective participation in research orientation; intellectual participation in the propaedeutic and/or therapeutic conduct of the studied cases; critical review of the manuscript.

Neusa Yuriko Sakai Valente: Approval of the final version of the manuscript; effective participation in research orientation; intellectual participation in the propaedeutic and/or therapeutic conduct of the studied cases; critical review of the manuscript.

## Conflicts of interest

None declared.
